# First Report of *Sarcocystis pilosa* from a Red Fox (*Vulpes vulpes*) Released for the Re-Introduction Project in South Korea

**DOI:** 10.3390/ani14010089

**Published:** 2023-12-27

**Authors:** Yeonghoon Jo, Sook Jin Lee, Mohammed Mebarek Bia, Seongjun Choe, Dong-Hyuk Jeong

**Affiliations:** 1 Laboratory of Wildlife Medicine, College of Veterinary Medicine, Chungbuk National University, Cheongju 28644, Republic of Korea; wildwildjo@chungbuk.ac.kr; 2 National Park Institute for Wildlife Conservation, Yeongju 36015, Republic of Korea; mfungus5@knps.or.kr; 3 Department of Parasitology, School of Medicine and Parasite Research Center, Chungbuk National University, Cheongju 28644, Republic of Korea; biamebarek@chungbuk.ac.kr; 4 The Wildlife Center of Chungbuk, Cheongju 28116, Republic of Korea

**Keywords:** 18S rRNA, cytochrome *c* oxidase subunit 1, host, red fox, restoration

## Abstract

**Simple Summary:**

In South Korea, the red fox (*Vulpes vulpes*) has been introduced and released as part of a restoration project since 2012. One of the released individuals was found dead two months after release in 2019. During necropsy, the intestinal contents were collected and oocysts of *Sarcocystis* sp. were found during coprological examination. PCR of the 18S rRNA gene and *cox1* gene sequences was conducted, and the isolate was identified as *Sarcocystis pilosa*. This is the first report of this species in South Korea, implying that there were mammals that originally acted as intermediate and definitive hosts.

**Abstract:**

The red fox (*Vulpes vulpes*) is a known host for various parasites, including numerous helminths and protozoans. Among these, certain species in the genus *Sarcocystis* (phylum Apicomplexa) have been documented to possess the capability to infect red foxes as definitive hosts. In South Korea, red foxes have been introduced and released as part of a re-introduction program. However, two months after its release, one of the foxes was found dead because of illegal trapping. The fox was necropsied, and a subsequent coprological study revealed oocysts of *Sarcocystis* sp. in the intestinal contents. The oocysts were identified as *Sarcocystis pilosa* based on the 18S rRNA and cytochrome *c* oxidase subunit 1 (*cox1*) gene sequences. It exhibited a 99.7–100% identity with 18S rRNA gene sequences and 99.1–99.8% identity with *cox1* gene sequences from other previously reported *S. pilosa* samples. Additionally, it showed identities of 95.4–96.4% and 91.1–91.5% with the *cox1* gene sequences of *S. hjorti* and *S. gjerdei*, while demonstrating 99.6 and 98.1% identity with the 18S rRNA gene sequences of *S. hjorti* and *S. gjerdei*, respectively. This is the first report from mainland Asia, excluding the Japanese archipelago, indicating that the life cycle of *S. pilosa* persists in South Korea.

## 1. Introduction

Red foxes (*Vulpes vulpes*) have stable populations in many countries. However, interestingly, they became completely extinct in Korea in the 1990s [1]. Accordingly, a red fox restoration project was initiated in 2012 as part of an effort to establish a stable population on the Korean Peninsula [2]. These efforts may be advantageous when using foxes as sentinel species for assessing ecosystems [3]. Meanwhile, the re-introduction of foxes also presents an intricate challenge to public health, given their susceptibility to a wide range of parasites, including zoonotic parasites such as *Cryptosporidium*, *Babesia*, *Trichinella*, *Toxocara*, and *Echinococcus*, among others [3]. Among these, the genus *Sarcocystis*, a protozoan parasite classified under the phylum Apicomplexa, has a broad host range and a two-host life cycle, with specificity that varies among species. Globally, this genus includes approximately 200 recognized species [4]. In South Korea, four *Sarcocystis* species have been identified among six mammalian host species [5,6,7,8,9,10]. Within the genus *Sarcocystis*, several species (such as *S. alces*, *S. capracanis*, *S. cruzi*, *S. gracilis*, *S. hjorti*, *S. pilosa*, *S. tenella*, etc.) are recognized for utilizing canids, including red fox, as their definitive host, while concurrently relying on ruminants (cervids, cattle, sheep, etc.) as their intermediate host. This has been observed in either natural or experimental settings in previous studies conducted in U.S.A., Australia, Europe and Japan [11,12,13,14,15].

The majority of *Sarcocystis* spp. rely on a predator–prey relationship, in which sarcocysts formed in the muscles of intermediate hosts are ingested by definitive hosts, leading to the development of oocysts/sporocysts in the intestinal mucosa of definitive hosts and their subsequent transmission through feces. Some species have been reported to utilize humans as either intermediate or definitive hosts [16]. To elucidate and address questions related to species and host identification of *Sarcocystis* spp., molecular methods, such as the analysis of 18S rRNA and *cox1* gene sequences, have become essential. Traditional methods based on parasite morphology and intermediate host identification can be limited and inconclusive. Specifically, 18S rRNA and *cox1* genes are ideal for this purpose due to their distinct advantages. The 18S rRNA gene is highly conserved across eukaryotes, enabling robust phylogenetic reconstruction and identification of closely related species [17], while the *cox1* gene has a faster rate of evolution compared with rRNA genes, offering higher resolution within closely related species [18]. These methods offer a more precise means of understanding the relationships between different species within the same clade. When *Sarcocystis* species are clustered together in phylogenetic trees based on genetic analyses, they often share common definitive hosts. This shared evolutionary history between the parasite and its host allows for the inference of definitive hosts, even for species with known intermediate hosts but unknown definitive hosts. This inference is based on the established definitive hosts of genetically related species [19].

*Sarcocystis pilosa* has been reported to utilize deer (*Cervus* spp.) as an intermediate host, and is predicted to utilize Canidae as a definitive host from a phylogenetic standpoint [20]. This prediction was confirmed as a fact when *S. pilosa* was identified in the feces of foxes (*Vulpes vulpes schrencki*) from Japan [15]. However, as there are currently no reports of *S. pilosa* in Korea, the primary objective of this study is to establish foundational information for monitoring diseases and parasites during the restoration of the red fox. This will be achieved by reporting and discussing the coincidental discovery of *S. pilosa* in Korea.

## 2. Materials and Methods

### 2.1. Animal and Sample Collection

An adult female fox, coded CF1624, was initially introduced from Northeast China to South Korea as part of a restoration project in 2018, following confirmation of its genetic identity as part of a group of Northeast Asian populations. Subsequently, it was released into the wild in South Korea in 2019 after a period of acclimation. Before being released into the wild, all introduced individuals were kept in outdoor enclosures of various sizes (100–8400 m^2^) in their untamed state. The foxes were provided with live chicks and chicken meat as food sources twice a day, with water provided *ad libitum*. All individuals were vaccinated for DHPPL (distemper, hepatitis, parainfluenza, parvo, and leptospirosis) and rabies and were administered anthelmintics four times during their stay in the enclosure. No significant findings were observed in pre-release fecal or blood examinations. CF1624 was found dead two months after being released because of illegal poaching. The movement of the individual was continuously monitored by GPS tracking, and the carcass was discovered a day after the movement stopped. During post-mortem examination to determine the cause of death and pathological peculiarities, the contents of the small intestine were collected and stored frozen at −20 °C. The weight of the individual before release was 6.16 kg, whereas that of the carcass was 4.84 kg.

### 2.2. Fecal Flotation and Cyst Observation

After fecal flotation using a saturated zinc sulfate solution, the supernatant was placed on a coverslip and observed under a light microscope (BX53; Olympus, Tokyo, Japan). Subsequently, the widths and lengths of the cystic structures were measured using the imaging software cellSens^®^ (version 3.2, Olympus cellSens software).

### 2.3. DNA Extraction and PCR Sequencing

The sample, diluted with PBS buffer, underwent three cycles of treatment: 10 min in a deep freezer and 3 min at 70 °C for each cycle. Subsequently, DNA was extracted using the QIAamp PowerFecal Pro DNA Kit^®^ (Qiagen, Hilden, Germany), following the manufacturer’s instructions. The concentration of extracted DNA was measured using a NanoDrop OneC spectrophotometer^®^ (Thermo Scientific, Waltham, MA, USA). The extracted DNA was subjected to PCR for the 18S rRNA gene (approximately 1600 bp) using the forward primer 1 L (5′-CCATGCATGTCTAAGTATAAGC-3′) [21] and the reverse primer R6 (5′-CGGAACACTCAATCGGTAGG-3′) [22] and for the cytochrome *c* oxidase subunit 1 gene (approximately 1000bp) using the forward primer SF1 (5′-ATGGCGTACAACAATCATAAAGAA-3′) [14] and the reverse primer SR9 (5′-ATATCCATACCRCCATTGCCCAT-3′) [23], utilizing the Mastercycler Nexus gradient^®^ (Eppendorf, Hamburg, Germany). Amplified DNA was sequenced by Cosmo Genetech (Seoul, South Korea).

### 2.4. Sequence and Phylogenetic Analysis

The 18S rRNA gene and *cox1* gene sequences obtained were used for phylogenetic analysis using MEGA X software version 10.0.5. For the 18S rRNA gene analysis, a set of four sequences was compiled from different geographical regions where *S. pilosa* was found, including South Korea, mainland Japan, Hokkaido, and Lithuania. Additionally, another set of 71 sequences was gathered, representing various *Sarcocystis* species and outgroup species sourced from different hosts. Notably, this compilation included sequences from *S. hjorti*, which is recognized as the species most closely related to *S. pilosa* [20].

For the analysis of the *cox1* gene, a collection of five sequences was assembled from diverse geographical locations where *S. pilosa* had been identified. These regions encompass South Korea, mainland Japan, Hokkaido, Lithuania, and Germany. Furthermore, we compiled an additional set of 35 sequences, focusing on *Sarcocystis* specimens collected from other canid fecal samples or *Sarcocystis* species recognized for utilizing canids as their definitive host. To broaden the scope, we incorporated *S. arctica* collected from canids’ muscle tissue, known for utilizing avians as their definitive host [24], to serve as an outgroup species. This dataset also encompassed *S. hjorti* and *S. gjerdei*, mirroring the inclusion in the 18S rRNA gene analysis.

Sequences were sourced from GenBank and aligned using the parameters specified in the ClustalW algorithm, which was integrated into the MEGA X software. The sequences used in the present study are provided in Table S1, which can be found in the Supplementary Materials.

To facilitate further analysis, minor truncations were applied to both ends of all sequences to ensure the preservation of homologous nucleotide positions. After truncation, the phylogenetic tree dataset ultimately consisted of sequences spanning 1248 positions for the 18S rRNA gene and 1002 positions for the *cox1* gene. To construct a phylogenetic tree, we chose the most suitable substitution model for sequence evolution using the jModelTest 2.1.10. In the analysis, we employed the optimal model GTR + I + G along with its corresponding parameters. A maximum likelihood (ML) tree was constructed using the PhyML 3.1/3.0 aLRT web server (http://www.phylogeny.fr/one_task.cgi?task_type=phyml, accessed on 25 October 2023, 14 December 2023) and subsequently evaluated using the bootstrap technique with 100 iterations for bootstrapping. The ultimate tree visualization was created using FigTree version 1.4.4. 18S rRNA gene sequences of *Sarcocystis pilosa* from mainland Japan, Hokkaido, and Lithuania, as well as *S. hjorti* and *S. gjerdei* sequences, were individually compared with sequences from South Korea. A similar comparison was carried out with *cox1* gene sequences. This was accomplished using the distance calculation function in MEGA X.

## 3. Results

### 3.1. Morphological Observations

**Figure 1:**
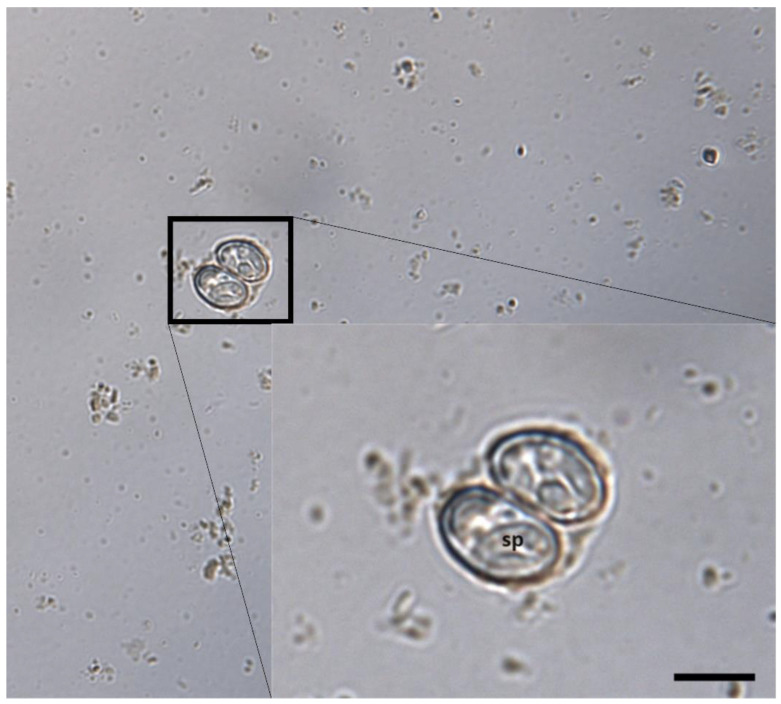
Morphology of a detected sporulated oocyst extracted from the feces of *Vulpes vulpes* under light microscopy. Scale bar: 10 μm; sp = sporozoite.

The cystic structures observed were *Sarcocystis* sporocysts, which had four sporozoites and were 16.0 (12.9~17.9) μm (SD = 0.9) long and 9.8 (7.0~13.8) μm (SD = 1.4) wide (mean values for 39 sporocysts). Some structures showed intact oocyst forms, which were composed of two sporocysts and a thin cyst wall (Figure 1).

### 3.2. Molecular Characteristics

The newly generated 18S rRNA gene sequence was 1095 bp in length and was submitted to GenBank (accession number: OR724702). The sequence exhibited 100% similarity to *S. pilosa* isolated from diaphragm muscles of Lithuanian sika deer (KU753891). Furthermore, the sequence displayed 100%, 100% and 99.7% similarity with samples obtained from diaphragm muscles of Hokkaido sika deer (LC466178), feces of Hokkaido red fox (LC496069) and muscles of sika deer in mainland Japan (LC349474), respectively. The sequences were 99.6 and 98.1% identical to the 18S rRNA gene sequences of *S. hjorti* (EU282017) and *S. gjerdei* (LC349475).

The newly generated *cox1* gene sequence was 1035 bp in length and was submitted to GenBank (accession number: OR947924). The acquired sequence showed a 99.7% similarity to *S. pilosa* identified in the diaphragm muscles of Lithuanian sika deer (KU753910) and a 99.8% similarity to that identified in the diaphragm muscles of German sika deer (OP617449). Additionally, it exhibited 99.8%, 99.7% and 99.1% similarity with specimens collected from Hokkaido (diaphragm muscles of sika deer: LC466201 and feces of red fox: LC496070) and mainland Japan (muscles of sika deer: LC349967), respectively. Furthermore, the sequences showed 95.4–96.4% and 91.1–91.5% identity with the *cox1* gene sequences of *S. hjorti* and *S. gjerdei*, respectively.

### 3.3. Phylogenetic Analysis

**Figure 2:**
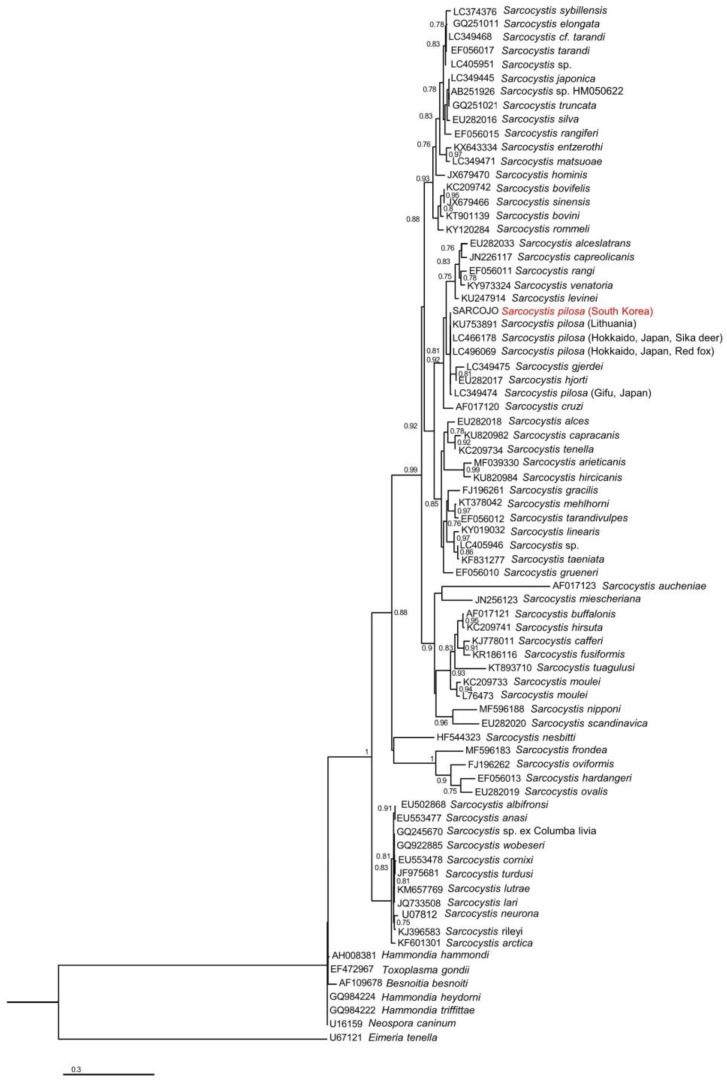
Maximum likelihood tree for *Sarcocystis* based on 18S rRNA gene sequences constructed using the GTR + I + G model. Bootstrap scores are expressed as proportion of 100 replications and are shown on each node. The sequence from the present study is highlighted in red. Node values less than 0.7 are not shown.

The acquired 18S rRNA gene sequence formed a monophyletic group with previously reported 18S rRNA gene sequences of *S. pilosa* in the phylogenetic tree (Figure 2). Nevertheless, this monophyletic group also encompasses *S. gjerdei* and *S. hjorti*, making it challenging to achieve a complete differentiation from *S. pilosa*.

**Figure 3:**
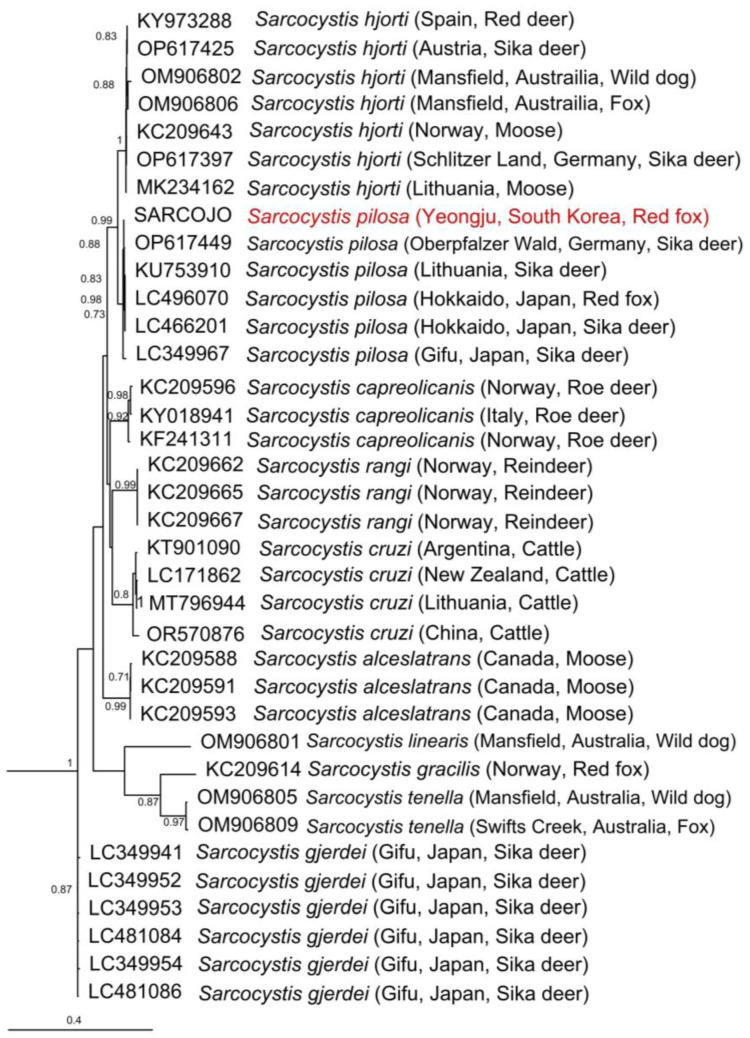
Maximum likelihood tree for *Sarcocystis* based on *cox1* gene sequences constructed using the GTR + I + G model. Bootstrap scores are expressed as proportion of 100 replications and are shown on each node. The sequence from the present study is highlighted in red. Node values less than 0.7 are not shown.

In Figure 3, the acquired *cox1* gene sequence formed a monophyletic group alongside previously reported *cox1* gene sequences of *S. pilosa*. Outgroup species (*Eimeria tenella*, *Sarcocystis arctica*) were excluded from the phylogenetic tree due to their considerable genetic divergence. The original tree is available in Figure S1 of the Supplementary Materials.

The distinction between *S. pilosa* and *S. hjorti* is more effectively accomplished through *cox1* gene sequences, as demonstrated in Prakas et al.’s study [20], rather than using 18S rRNA gene sequences.

## 4. Discussion

Sarcocystis infections such as *S. cruzi*, *S. miescheriana*, *S. tenella*, and *S. grueneri* have been reported from cattle (*Bos taurus coreanae*), pigs (*Sus scrofa domesticus*), goats (*Capra hircus coreanae*), red deer (*Cervus elaphus*), Korean water deer (*Hydropotes inermis argyropus*), and striped field mice (*Apodemus agrarius*) in South Korea [5,6,7,8,9,10]. These infected animals are intermediate hosts, and no natural infection with *Sarcocystis* has been reported in definitive hosts in South Korea, except for experimental infections conducted in dogs with *S. cruzi* [6]. In the present study, we confirmed that *Sarcocystis* sp. naturally infect red foxes in South Korea. Based on the 18S rRNA sequence, it was identified as *S. pilosa*. This is the first report of its definitive host on the main landmass of Eurasian continent. Previously reported definitive hosts of *S. pilosa* have been limited to a single fox species in Hokkaido, Japan, as in the present study. *Sarcocystis pilosa* has been previously documented in intermediate hosts such as sika deer (*Cervus nippon*) and red deer (*C. elaphus*), with confirmed occurrences in Lithuania, Japan, Switzerland, and Germany [20,25,26,27]. In the constructed phylogenetic tree, we confirmed that the sequences obtained in this study formed a clade with previously reported sequences of *S. pilosa*. As this was not a full-sequence analysis, it was challenging to identify significant sequence differences based on geographical distribution. However, we have confirmed the genetic similarity of *S. pilosa*, indicating its possible distribution across the Eurasian region.

*Sarcocystis* infection in this red fox may have originated from the source population in China before its translocation to Korea, but it also could have originated from local prey sources after its release. Considering the latent period within the definitive hosts of *Sarcocystis*, it takes approximately 14 days post-infection for oocysts to become fully sporulated in closely related species, such as *S. alces* and *S. hjorti* [13]. However, regarding the shedding period (patent period) in *S. falcatula* using Virginia opossums (*Didelphis virginiana*) as definitive hosts, shedding continued until euthanasia of all infected individuals (46–200 days post-infection) [28]. Similarly, in other studies involving dogs and cats experimentally infected with *Sarcocystis* species utilizing various intermediate hosts, such as cattle, horses, pigs, and sheep, shedding persisted until euthanasia, making it challenging to precisely determine the common shedding period of *Sarcocystis* [29]. Therefore, while it is not possible to completely exclude either of the two hypotheses, it is noteworthy that when *Sarcocystis* sp. utilizing guanacos (*Lama guanicoe*) as an intermediate host was experimentally induced in dogs, an average patent period of 45.6 days (19–61 days) was observed [30]. Additionally, experimental infections with *S. tenella* in dogs and red foxes have been reported to result in sporocyst counts of 100 or fewer per gram of feces at 60 days post-infection [12]. Considering the absence of significant findings related with internal parasite infection in the pre-release fecal examination (microscopical observation after flotation and sedimentation method) and release one year after introduction from China, it appears unlikely that the individual was infected at the time of introduction. Additionally, considering the spectrum of intermediate hosts for *S. pilosa* and closely related species, under the assumption of post-release infection from local prey sources, the reported intermediate hosts were limited to cervids, with no records beyond that.

Four native cervid species naturally coexist on the Korean Peninsula: roe deer (*Capreolus pygargus*), musk deer (*Moschus moschiferus*), sika deer (*Cervus nippon*), and water deer (*H. inermis argyropus*). Among these, the sika deer is the sole reported intermediate host of *S. pilosa*. Indeed, foxes have been observed opportunistically scavenging on cervid carcasses as a food source [31], and beyond the sika deer, other cervid species, like water deer, also hold the potential to act as intermediate hosts. Therefore, there is a need to confirm the presence of *S. pilosa* from these hosts. Furthermore, considering the proximity of the dead fox’s activity area to deer farms where captive cervids (*Cervus nippon*, *C. elaphus*, *C. canadensis*) are raised, it is essential to investigate the potential infection of captive deer by *Sarcocystis* species. Additionally, it is important to ascertain which canid species served as definitive hosts before the introduction of foxes for restoration. Excluding domestic dogs, raccoon dogs (*Nyctereutes procyonoides*) are the most prevalent species among the indigenous wild canids of the Korean Peninsula. Experimental evidence has revealed its potential role as a definitive host for *Sarcocystis* species [32]. Consequently, future research employing fecal analyses should be undertaken to determine the occurrence of *Sarcocystis* infections in raccoon dogs. Such investigations would greatly aid in unraveling the ecological circulation pathways of *Sarcocystis* in domestic landscapes.

*Sarcocystis* infection in definitive hosts causes symptoms that are generally mild or asymptomatic and are regarded as less pathogenic. In humans, clinical symptoms such as stomach aches, nausea, and diarrhea have been reported in cases of intestinal sarcocystosis [10]. In other definitive host species, especially canines and felines, most infected individuals appear asymptomatic or may show acute-to-chronic diarrhea. However, an unusual case of megacolon and amyloidosis due to chronic inflammation induced by *Sarcocystis* sp. infection has been reported in a dog [33]. In the present study, the deceased red fox exhibited gastric perforation, intraperitoneal leakage of gastric contents, and constriction of the rectum and bladder neck. The bladder was congested with blood, and the collected intestinal contents displayed slight mucoid features. However, no histological analysis was conducted, making it difficult to correlate these findings directly with *Sarcocystis* infection.

From a One Health perspective, *Sarcocystis* typically demonstrates a generalized pattern of infecting related host species within its host range. Although the potential of *S. pilosa*, which utilizes ruminants as intermediate hosts, to infect humans seems low owing to this common pattern, it has not been experimentally confirmed. However, when non-human *Sarcocystis* spp. sporocysts are accidentally ingested, humans can also become dead-end hosts (aberrant intermediate hosts), exhibiting possible clinical symptoms of extraintestinal sarcocystosis ranging from asymptomatic muscle cysts to a severe, sudden-onset eosinophilic myositis accompanied by systemic symptoms and blood eosinophilia [10]. Therefore, it is essential to consider concerns related to water or food contamination from wildlife feces in the context of this issue.

## 5. Conclusions

In the present study, we confirm that, as previously reported, red foxes can serve as definitive hosts for *S. pilosa* in their natural environment. Additionally, we report the presence of *S. pilosa* in Korea, a country that is geographically close to Japan and part of continental Asia. Furthermore, this study indirectly highlights the potential intermediate hosts of *S. pilosa* and suggests the presence of mammalian species that previously acted as definitive hosts before the introduction of foxes to the Korean Peninsula. This highlights the direction for future research, including the confirmation and reporting of existing natural hosts of *S. pilosa* in South Korea. In addition, it suggests that conservation programs to restore ecosystem health may further accelerate the spread of diseases, highlighting the need to recognize the importance of disease epidemiology and quarantine, as well as the ecological implications, in this type of re-introduction program.

## Data Availability

Data is contained within the article and Supplementary Materials.

## Supplementary Materials

The following supporting information can be downloaded at: https://www.mdpi.com/article/10.3390/ani14010089/s1, Table S1: List of sequences included in the phylogenetic analyses with details of collection localities, host species and GenBank accession numbers; Figure S1: Original phylogenetic tree of *Sarcocystis* spp. based on the *cox1* gene.
