# Supplementary material for: First Report of Sarcocystis pilosa from a Red Fox (Vulpes vulpes) Released for the Re-Introduction Project in South Korea
Source: Animals (Basel). 2023 Dec 27;14(1):89. doi: 10.3390/ani14010089 (PMC10778215; doi:10.3390/ani14010089)
Supplement: Supplementary file 1 [file animals-14-00089-s001.zip › Table S1 List of sequences included in the phylogenetic analyses with details of collection localities, host species and GenBank accession numbers.pdf]

Table S1. List of sequences included in the phylogenetic analyses with details of collection localities, host species and GenBank accession numbers

Number*	Parasite species (gene)	Host species	Locality	GenBank accession no.
	**Eimeriidae (18S rRNA)**			
1	*Eimeria tenella*	Chicken (Unspecified)	N/A	U67121
	**Sarcocystidae (18S rRNA)**			
2	*Besnoitia besnoiti*	Cattle *(Bos taurus)*	Republic of South Africa	AF109678
3	*Hammondia hammondi*	Tasmanian Bennetts wallabies (*Macropus rufogriseus*)	Australia (Tasmania)	AH008381
4	*Hammondia heydorni*	Dog (*Canis lupus familiaris*)	Norway	GQ984224
5	*Hammondia triffittae*	Red fox (*Vulpes vulpes*)	Norway	GQ984222
6	*Neospora caninum*	Dog (*Canis familiaris*)	Sweden	U16159
7	*Sarcocystis arctica*	Arctic fox (*Vulpes lagopus*)	Norway	KF601301
8	*Sarcocystis albifronsi*	White–fronted geese (*Anser albifrons*)	Lithuania	EU502868
9	*Sarcocystis alces*	Moose (*Alces alces*)	Norway	EU282018
10	*Sarcocystis alceslatrans*	Moose (*Alces alces*)	Canada	EU282033
11	*Sarcocystis anasi*	Hooded crow (*Corvus cornix*)	Lithuania (Vilnius and Šilutė)	EU553477
12	*Sarcocystis arieticanis*	Sheep (*Ovis aries*)	China	MF039330
13	*Sarcocystis aucheniae*	Alpaca (*Vicugna pacos*)	Australia	AF017123
14	*Sarcocystis bovifelis*	Cattle (*Bos taurus*)	Argentina	KC209742

15	*Sarcocystis bovini*	Cattle (*Bos taurus*)	Argentina	KT901139
16	*Sarcocystis buffalonis*	Water buffalo (*Bubalus bubalis*)	Vietnam	AF017121
17	*Sarcocystis cafferi*	African buffalo (*Syncerus caffer*)	Pretoria	KJ778011
18	*Sarcocystis capracanis*	Goat (*Capra hircus*)	China	KU820982
19	*Sarcocystis capreolicanis*	Roe deer (*Capreolus capreolus*)	Norway	JN226117
20	*Sarcocystis cf. tarandi*	Sika deer (*Cervus nippon centralis*)	Japan	LC349468
21	*Sarcocystis cornixi*	Hooded crow (*Corvus cornix*)	Lithuania	EU553478
22	*Sarcocystis cruzi*	Cattle (*Bos taurus*)	Sweden	AF017120
23	*Sarcocystis elongata*	Red deer (*Cervus elaphus*)	Norway	GQ251011
24	*Sarcocystis entzerothi*	Roe deer (*Capreolus capreolus*)	Lithuania	KX643334
25	*Sarcocystis frondea*	Sika deer (*Cervus nippon*)	Lithuania	MF596183
26	*Sarcocystis fusiformis*	Water buffalo (*Bubalus bubalis*)	Egypt	KR186116
27	*Sarcocystis gigantea*	Sheep (*Ovis aries*)	Norway	KC209733
28	*Sarcocystis gjerdei*	Sika deer (*Cervus nippon centralis*)	Japan	LC349475
29	*Sarcocystis gracilis*	Roe deer (*Capreolus capreolus*)	Norway	FJ196261
30	*Sarcocystis grueneri*	Reindeer (*Rangifer tarandus*)	Norway	EF056010
31	*Sarcocystis hardangeri*	Reindeer (*Rangifer tarandus*)	Norway	EF056013

32	*Sarcocystis hircicanis*	Goat (*Capra hircus*)	China	KU820984
33	*Sarcocystis hirsuta*	Cattle (*Bos taurus*)	Argentina	KC209741
34	*Sarcocystis hjorti*	Moose (*Alces alces*)	Norway	EU282017
35	*Sarcocystis hominis*	Cattle (*Bos taurus*)	Argentina	JX679470
36	*Sarcocystis japonica*	Sika deer (*Cervus nippon centralis*)	Japan	LC349445
37	*Sarcocystis lari*	Great black–backed gull (*Larus marinus*)	Lithuania	JQ733508
38	*Sarcocystis levinei*	Water buffalo (*Bubalus bubalis*)	Egypt	KU247914
39	*Sarcocystis linearis*	Roe deer (*Capreolus capreolus*)	Italy	KY019032
40	*Sarcocystis lutrae*	Eurasian otter (*Lutra lutra*)	Norway	KM657769
41	*Sarcocystis matsuoae*	Sika deer (*Cervus nippon centralis*)	Japan	LC349471
42	*Sarcocystis mehlhorni*	Black–tailed deer (*Odocoileus hemionus columbianus*)	USA	KT378042
43	*Sarcocystis miescheriana*	Wild boar (*Sus scrofa*)	Lithuania	JN256123
44	*Sarcocystis moulei*	Goat (*Capra hircus*)	Afghanistan	L76473
45	*Sarcocystis nesbitti*	Human (*Homo sapiens*)	Malaysia	HF544323
46	*Sarcocystis neurona*	Brown–headed cowbirds (*Molothrus ater*)	USA	U07812
47	*Sarcocystis nipponi*	Sika deer (*Cervus nippon*)	Lithuania	MF596188
48	*Sarcocystis ovalis*	Moose (*Alces alces*)	Norway	EU282019

49	*Sarcocystis oviformis*	Roe deer (*Capreolus capreolus*)	Norway	FJ196262
50	*Sarcocystis pilosa*	Sika deer (*Cervus nippon yesoensis*)	Japan (Hokkaido)	LC466178
51	*Sarcocystis pilosa*	Red fox (*Vulpes vulpes schrencki*)	Japan (Hokkaido)	LC496069
52	*Sarcocystis pilosa*	Sika deer (*Cervus nippon*)	Lithuania	KU753891
53	*Sarcocystis pilosa*	Sika deer (*Cervus nippon centralis*)	Japan (Gifu)	LC349474
54	*Sarcocystis rangi*	Reindeer (*Rangifer tarandus tarandus*)	Norway	EF056011
55	*Sarcocystis rangiferi*	Reindeer (*Rangifer tarandus tarandus*)	Norway	EF056015
56	*Sarcocystis rileyi*	Common eider (*Somateria mollissima*)	Norway	KJ396583
57	*Sarcocystis rommeli*	Cattle (*Bos taurus*)	China	KY120284
58	*Sarcocystis scandinavica*	Moose (*Alces alces*)	Norway	EU282020
59	*Sarcocystis silva*	Moose (*Alces alces*)	Norway	EU282016
60	*Sarcocystis sinensis*	Cattle (*Bos taurus*)	Argentina	JX679466
61	*Sarcocystis sp.*	Domestic pigeon (*Columba livia f. domestica*)	Germany	GQ245670
62	*Sarcocystis sp.*	Sika deer (*Cervus nippon*)	Japan	LC405946
63	*Sarcocystis sp.*	Sika deer (*Cervus nippon*)	Japan	LC405951
64	*Sarcocystis sp.*	Sika deer (*Cervus nippon yesoensis*)	Japan	AB251926
65	*Sarcocystis sybillensis*	Sika deer (*Cervus nippon*)	Japan	LC374376

66	*Sarcocystis taeniata*	Moose (*Alces alces*)	Canada	KF831277
67	*Sarcocystis tarandi*	Reindeer (*Rangifer tarandus tarandus*)	Norway	EF056017
68	*Sarcocystis tarandivulpes*	Reindeer (*Rangifer tarandus tarandus*)	Norway	EF056012
69	*Sarcocystis tenella*	Sheep (*Ovis aries*)	Norway	KC209734
70	*Sarcocystis truncata*	Red deer (*Cervus elaphus*)	Norway	GQ251021
71	*Sarcocystis tuagulusi*	Williamson's mouse deer (*Tuagulus williamsoni*)	China	KT893710
72	*Sarcocystis turdusi*	Blackbird (*Turdus merula*)	Lithuania	JF975681
73	*Sarcocystis venatoria*	Red deer (*Cervus elaphus*)	Spain	KY973324
74	*Sarcocystis wobeseri*	Mallard duck (*Anas platyrhynchos*)	Lithuania	GQ922885
75	*Toxoplasma gondii*	N/A	China	EF472967
	**Eimeriidae (*cox1*)**			
76	*Eimeria tenella*	Chicken (Unspeicifed)	N/A	HG994976
	**Sarcocystidae (*cox1*)**			
77	*Sarcocystis alcestrans*	Mooses (*Alces alces*)	Canada	KC209591
78	*Sarcocystis alcestrans*	Mooses (*Alces alces*)	Canada	KC209593
79	*Sarcocystis alcestrans*	Mooses (*Alces alces*)	Canada	KC209588
80	*Sarcocystis arctica*	Red fox (*Vulpes vulpes*)	Czech Republic	KY609324
81	*Sarcocystis arctica*	Red fox (*Vulpes vulpes*)	Latvia	MF596289
82	*Sarcocystis arctica*	Red fox (*Vulpes vulpes*)	Spain	MF596306
83	*Sarcocystis arctica*	Arctic fox (*Vulpes lagopus*)	Norway	KF601325

84	*Sarcocystis capreolicanis*	Roe deer (*Capreolus capreolus*)	Norway	KF241311
85	*Sarcocystis capreolicanis*	Roe deer (*Capreolus capreolus*)	Norway	KC209596
86	*Sarcocystis capreolicanis*	Roe deer (*Capreolus capreolus*)	Italy	KY018941
87	*Sarcocystis cruzi*	Cattle (*Bos taurus*)	Lithuania	MT796944
88	*Sarcocystis cruzi*	Cattle (*Bos taurus*)	Argentina	KT901090
89	*Sarcocystis cruzi*	Cattle (*Bos taurus*)	New Zealand	LC171862
90	*Sarcocystis cruzi*	Cattle (*Bos taurus*)	China	OR570876
91	*Sarcocystis gjerdei*	Sika deer (*Cervus nippon centralis*)	Japan (Gifu)	LC349941
92	*Sarcocystis gjerdei*	Sika deer (*Cervus nippon centralis*)	Japan (Gifu)	LC349952
93	*Sarcocystis gjerdei*	Sika deer (*Cervus nippon centralis*)	Japan (Gifu)	LC349953
94	*Sarcocystis gjerdei*	Sika deer (*Cervus nippon centralis*)	Japan (Gifu)	LC349954
95	*Sarcocystis gjerdei*	Sika deer (*Cervus nippon centralis*)	Japan (Gifu)	LC481084
96	*Sarcocystis gjerdei*	Sika deer (*Cervus nippon centralis*)	Japan (Gifu)	LC481086
97	*Sarcocystis gracilis*	Red fox (*Vulpes vulpes*)	Norway	KC209614
98	*Sarcocystis hjorti*	Moose (*Alces alces*)	Norway	KC209643
99	*Sarcocystis hjorti*	Moose (*Alces alces*)	Lithuania	MK234162
100	*Sarcocystis hjorti*	Red deer (*Cervus elaphus*)	Spain	KY973288
101	*Sarcocystis hjorti*	Sika deer (*Cervus nippon*)	Germany (Schlitzer Land)	OP617397
102	*Sarcocystis hjorti*	Sika deer (*Cervus nippon*)	Austria (Tullner Donauauen)	OP617425
103	*Sarcocystis hjorti*	Wild dog (Unspecified)	Australia (Mansfield)	OM906802
104	*Sarcocystis hjorti*	Fox (Unspecified)	Australia (Mansfield)	OM906806
105	*Sarcocystis linearis*	Wild dog (Unspecified)	Australia (Mansfield)	OM906801

106	*Sarcocystis pilosa*	Sika deer (*Cervus nippon yesoensis*)	Japan (Hokkaido)	LC466201
107	*Sarcocystis pilosa*	Red fox (*Vulpes vulpes schrencki*)	Japan (Hokkaido)	LC496070
108	*Sarcocystis pilosa*	Sika deer (*Cervus nippon centralis*)	Japan (Gifu)	LC349967
109	*Sarcocystis pilosa*	Sika deer (*Cervus nippon*)	Lithuania	KU753910
110	*Sarcocystis pilosa*	Sika deer (*Cervus nippon*)	Germany (Oberpfalzer Wald)	OP617449
111	*Sarcocystis rangi*	Reindeer (*Rangifer tarandus*)	Norway	KC209662
112	*Sarcocystis rangi*	Reindeer (*Rangifer tarandus*)	Norway	KC209665
113	*Sarcocystis rangi*	Reindeer (*Rangifer tarandus*)	Norway	KC209667
114	*Sarcocystis tenella*	Wild dog (Unspecified)	Australia (Mansfield)	OM906805
115	*Sarcocystis tenella*	Fox (Unspecified)	Australia (Swifts Creek)	OM906809
