# Supplementary figures and images for: First Report of Sarcocystis pilosa from a Red Fox (Vulpes vulpes) Released for the Re-Introduction Project in South Korea
Source: Animals (Basel). 2023 Dec 27;14(1):89. doi: 10.3390/ani14010089 (PMC10778215; doi:10.3390/ani14010089)

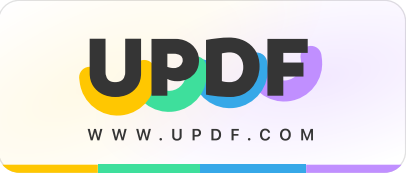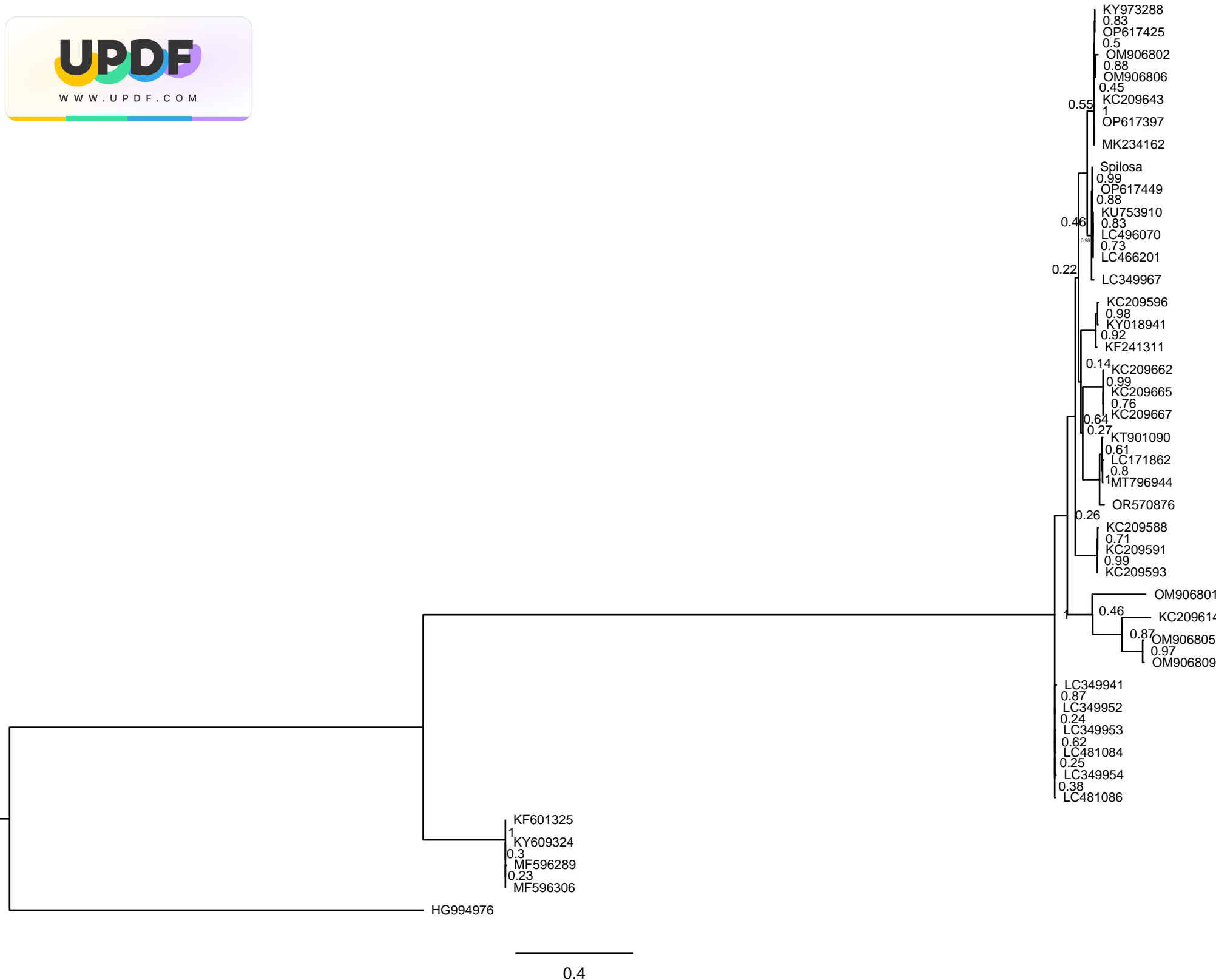

Supplement: Supplementary file 1 [file animals-14-00089-s001.zip › Figure S1 Original phylogenetic tree of Sarcocystis spp. based on cox1 gene.pdf]
